# Non-alcoholic fatty liver disease is a strong predictor of coronary artery calcification in metabolically healthy subjects: A cross-sectional, population-based study in middle-aged subjects

**DOI:** 10.1371/journal.pone.0202666

**Published:** 2018-08-22

**Authors:** Anders Gummesson, Ulf Strömberg, Caroline Schmidt, Joel Kullberg, Oskar Angerås, Stefan Lindgren, Ola Hjelmgren, Kjell Torén, Annika Rosengren, Björn Fagerberg, John Brandberg, Göran Bergström

**Affiliations:** 1 Department of Clinical Pathology and Genetics, Sahlgrenska University Hospital, Gothenburg, Sweden; 2 Department of Molecular and Clinical Medicine, Sahlgrenska Academy, University of Gothenburg, Gothenburg, Sweden; 3 Health Metrics Unit, Institute of Medicine, Sahlgrenska Academy, University of Gothenburg, Gothenburg, Sweden; 4 Department of Surgical Sciences, Section of Radiology, Uppsala University, Uppsala, Sweden; 5 Antaros Medical, Mölndal, Sweden; 6 Department of Cardiology, Sahlgrenska University Hospital, Gothenburg, Sweden; 7 Gastroenterology Division, Department of Clinical Sciences, Lund University, University Hospital Skåne, Malmö, Sweden; 8 Clinical Physiology, Sahlgrenska University Hospital, Gothenburg, Sweden; 9 Section of Occupational and Environmental Medicine, Sahlgrenska Academy, University of Gothenburg, Gothenburg, Sweden; 10 Department of Radiology, Sahlgrenska University Hospital, Gothenburg, Sweden; Medizinische Fakultat der RWTH Aachen, GERMANY

## Abstract

**Objectives:**

This study aims to estimate the relationship between non-alcoholic fatty liver disease (NAFLD) and measures of atherosclerotic cardiovascular disease (ASCVD), and to determine to what extent such relationships are modified by metabolic risk factors.

**Methods:**

The study was conducted in the population-based Swedish CArdioPulmonary bioImage Study (SCAPIS) pilot cohort (n = 1015, age 50–64 years, 51.2% women). NAFLD was defined as computed tomography liver attenuation ≤40 Hounsfield Units, excluding other causes of liver fat. Coronary artery calcification score (CACS) was assessed using the Agatston method. Carotid plaques and intima media thickness (IMT) were measured by ultrasound. Metabolic status was based on assessments of glucose homeostasis, serum lipids, blood pressure and inflammation. A propensity score model was used to balance NAFLD and non NAFLD groups with regards to potential confounders and associations between NAFLD status and ASCVD variables in relation to metabolic status were examined by logistic and generalized linear regression models.

**Results:**

NAFLD was present in 106 (10.4%) of the subjects and strongly associated with obesity-related traits. NAFLD was significantly associated with CACS after adjustment for confounders and metabolic risk factors (OR 1.77, 95% CI 1.07–2.94), but not with carotid plaques and IMT. The strongest association between NAFLD and CACS was observed in subjects with few metabolic risk factors (n = 612 [60% of all] subjects with 0–1 out of 7 predefined metabolic risk factors; OR 5.94, 95% CI 2.13–16.6).

**Conclusions:**

NAFLD was independently associated with coronary artery calcification but not with measures of carotid atherosclerosis in this cohort. The association between NAFLD and CACS was most prominent in the metabolically healthy subjects.

## Introduction

Non-alcoholic fatty liver disease (NAFLD) represents a spectrum of liver conditions ranging from isolated fatty liver (steatosis) to fatty liver along with inflammation (i.e. non-alcoholic steatohepatitis [NASH]). Although NAFLD can progress to liver failure and hepatocellular carcinoma [1, 2], the most common cause of death in NAFLD patients is related to atherosclerotic cardiovascular disease (ASCVD) [3] and a large number of studies have shown that NAFLD is a significant risk factor for atherosclerosis in the coronary and carotid arteries [4–17]. Obesity is a major cause of NAFLD and along with the increasing prevalence of obesity, NAFLD has become the most common liver disease with an estimated prevalence of 25% globally [2]. Most NAFLD patients also display other obesity-related metabolic aberrations such as impaired glucose homeostasis, dyslipidemia, hypertension, and low-grade inflammation [2]. These metabolic risk factors could potentially explain the increased incidence of ASCVD in NAFLD patients and current clinical practice guidelines for the management of NAFLD recommends that all individuals with steatosis should be screened for features of the metabolic syndrome (MetS) [18]. Given the central role of metabolic risk factors in the management of NAFLD, it is important to understand how these factors modify the relationship between NAFLD and ASCVD and more specifically to assess cardiovascular risk for patients who appear metabolically unaffected by their NAFLD as judged from clinical parameters. This aspect is important as it could have implications for the medical management of this patient population with regards to ASCVD surveillance and prevention. To our knowledge, few studies have addressed this question specifically. Subgroup analyses in one large Asian study indicated that NAFLD assessed by ultrasound was a stronger risk factor for coronary artery disease in MetS than in non-MetS subjects [14]. On the other hand, another study reported that NAFLD assessed by ultrasound was associated with carotid intima-media thickness only in MetS and not in non-MetS subjects [19]. There is an inherent problem with grouping based on MetS in that the established definitions include obesity criteria, which makes it difficult to dissect obesity-driven metabolic derangements from obesity *per se*. Given the strong effect of obesity on liver fat as well as on a wide range of metabolic risk factors, results can be difficult to interpret if comparison groups are not balanced with regards to adiposity measures. Also, current MetS definitions do not include metabolic aberrations such as hyperinsulinemia and inflammation which theoretically might modify the relationship between NAFLD and ASCVD.

In the current study we investigated the relationships between NAFLD, assessed by computed tomography (CT), and measures of atherosclerosis in the coronary and carotid arteries in middle-aged subjects in the population-based Swedish CArdioPulmonary bioImage Study (SCAPIS) pilot cohort. The main aim was to investigate the modifying effects of a wide range of obesity-driven metabolic risk factors, using the odds ratio as the effect parameter for associations between NAFLD and dichotomized ASCVD measures and the ratio of means as the effect parameter for associations between NAFLD and continuous ASCVD measures, and to address this aim we used a propensity score model to balance NAFLD and non-NAFLD groups with regards to obesity measurements and potential confounders.

## Methods

### Study design and population

The SCAPIS pilot study is a population-based, cross-sectional study conducted at the Sahlgrenska University Hospital in Göteborg, Sweden [20]. In 2012, a randomly selected population sample including 2243 Swedish adults aged 50 to 65 years were recruited from the census register, stratified for low and high socioeconomic status area. Out of these, 1111 (49.5%) agreed to participate. During two days, the participants underwent extensive imaging and functional studies of the heart, lungs and metabolism. They also completed an extensive questionnaire regarding lifestyle and living conditions. The study is approved by the Ethical Review Board of Umeå, Sweden (Dnr 2010-228-31), within the framework of the national SCAPIS project. All participants provided written informed consent. The study protocol conforms to the ethical guidelines of the 1975 Declaration of Helsinki.

**Fig 1:**
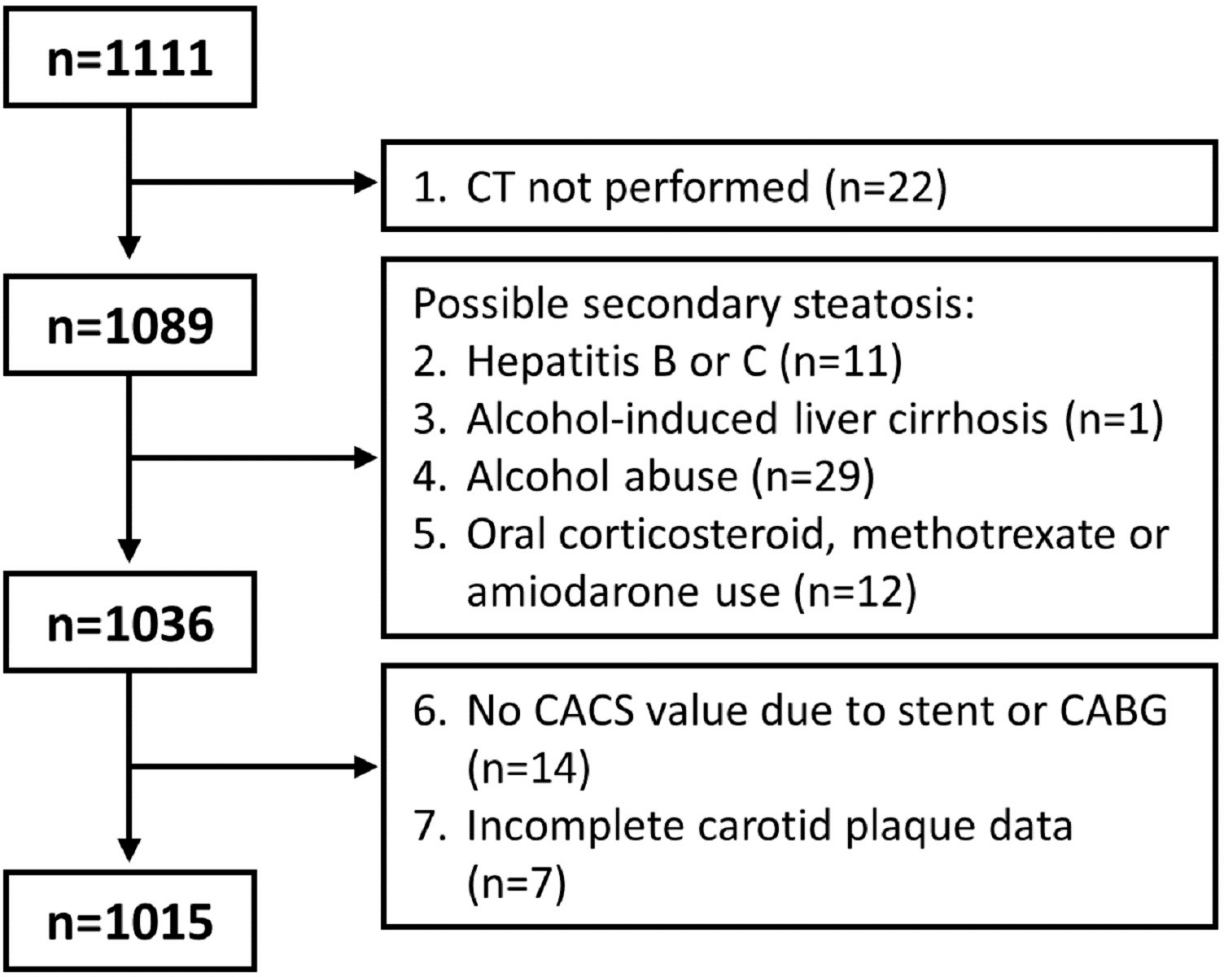
Reasons for excluding SCAPIS pilot participants in the present study.

Among the 1111 participants, 1089 underwent CT imaging to assess fatty liver (Fig 1). A total of 53 participants were excluded for possible secondary hepatic steatosis, including chronic hepatitis, alcohol-induced liver disease according to medical history, high alcohol consumption according to questionnaires (alcohol intake of 20 g/day or more in women and 30 g/day or more in men, or a total Alcohol Use Disorder Identification Test [AUDIT][21] score of 16 or more), or oral corticosteroid, methotrexate or amiodarone use. Finally, 21 participants were excluded from the dataset due to missing CACS or carotid plaque data.

### Computed tomography imaging

Imaging for coronary artery calcification score (CACS) and fat depots (liver and abdominal) was performed using a dedicated dual-source CT scanner equipped with a Stellar Detector (Siemens, Somatom Definition Flash, Siemens Medical Solution, Forchheim, Germany) as previously described [20]. Liver and abdominal adipose tissues were assessed using single slice techniques in combination with fully automated image analysis [22]. The slice thickness used was 5 mm and the liver slice covered both lobes and the spleen and the abdominal slice was acquired at the L4-level. Hepatic steatosis was assessed as mean liver attenuation in a large automatically segmented region of the liver. NAFLD was defined as liver attenuation ≤40 Hounsfield Units (HU) [23]. Abdominal adipose tissue depots were separated into visceral and subcutaneous adipose tissue by an automated algorithm. The fully automated analysis has been validated by comparisons to manual reference measurements (n = 50, unpublished data) and achieved correlation coefficient of 0.997, 0.998, and 0.999 for liver attenuation, visceral, and subcutaneous areas, respectively [22].

The calcium content in each coronary artery was measured and summed to produce a total coronary artery calcification score (CACS) according to McCollough [24] and Agatston [25]. A subset of the subjects with positive CACS were imaged using 100 kV (n = 84). CACS from these subjects have been recalculated to the standard 120 kV as described by Deprez *et al* [26]. In repeat reading of images from 50 randomly selected subjects the kappa measure of agreement [27] was 0.91 in identifying subjects with >0 in CACS.

### Ultrasound imaging

Ultrasound examination was performed with an ultrasound scanner (SIEMENS S2000) equipped with a linear transducer 9L4. An ECG signal (lead II) was simultaneously recorded to be able to save a frozen image on top of the R-wave to minimize variability. The right and left carotid arteries were scanned from the distal part of the common carotid artery and approximately 10 mm upstream in the internal and external carotid arteries with the aim to identify and record the occurrence of atherosclerotic plaques. Images for measurement of IMT were recorded from the far wall in the CCA and the carotid bulb. At the position of the thickest part (visually judged), two separately frozen longitudinal images were saved. One motion image loop was saved to assist in interpreting the frozen images [28]. A plaque was considered present if it met the Mannheim plaque consensus [29]. Images were measured in an analyzing system based on automated detection of the echo structures, with the option to make manual corrections by the operator. The average thickness of the intima-media complex for 10-mm long sections of the common carotid artery and the carotid bulb, respectively were measured. Reproducibility of plaque measurements and IMT in our laboratory has previously been published [30, 31].

### Covariate assessments

Information on education level, alcohol, smoking and physical activity was collected through self-administrated questionnaires. Weight was measured with participants in light clothing, using calibrated scales. The body mass index (BMI) was calculated by dividing the weight (kg) by the square of the height (m). Blood pressure was calculated as the average of three measurements, after 5-minute supine rest, measured in the right brachial artery. Hypertension was defined as self-reported use of antihypertensive medications, or systolic blood pressure ≥140 or diastolic blood pressure ≥90 mmHg [32]. Diabetes was defined as self-reported diagnosis of diabetes including antidiabetic medication, or fasting plasma glucose ≥7.0 mmol/L at SCAPIS Pilot visit 1 and visit 2, or Hemoglobin A1c (HbA1c) ≥48 mmol/mol at visit 1. Impaired fasting glucose (IFG) was defined as fasting plasma glucose measured ≥6.1 mmol/L [33] at visit 1, high triglyceride levels as serum triglycerides ≥1.7 mmol/L (150 mg/dl), low HDL levels as serum HDL-cholesterol <1.3 mmol/L (50 mg/dl) in women and <1.0 mmol/L (40 mg/dl) in men, abdominal obesity as a waist circumference ≥102 cm in men and ≥88 cm in women, high insulin as a serum insulin ≥20 mU/L, high LDL as ≥4.0 mmol/L (≥155 mg/dl), and low-grade inflammation as high-sensitivity C-reactive protein (hsCRP) ≥5 mg/L.

### Statistical analysis

**Fig 2:**
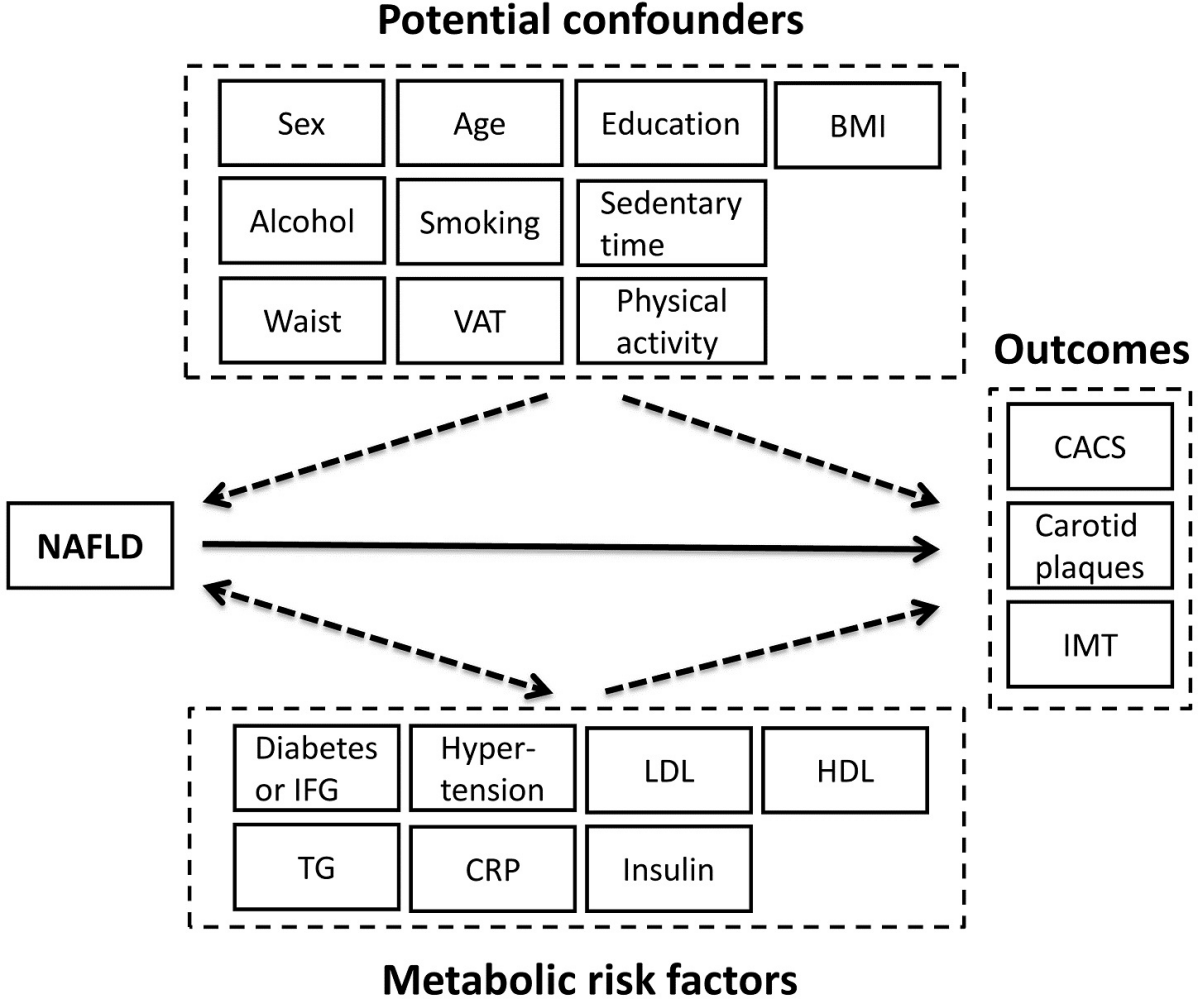
Schematic illustration of an underlying, hypothetical causal model framework considered when modelling cross-sectional relationships between NAFLD, confounders, metabolic risk factors, and outcome measures of atherosclerotic cardiovascular disease. NAFLD is the risk factor studied. The potential confounders may influence NAFLD but not vice versa. By contrast, the metabolic risk factors may be influenced or modified by NAFLD, i.e. some of these factors may act as intermediates in the potential causal pathways between NAFLD and the outcomes.

The statistical analysis aimed at examining associations based on a model framework for underlying relationships between NAFLD, confounders, metabolic risk factors and outcomes (CACS, carotid plaques and IMT) (Fig 2). NAFLD should not influence or modify any of the variables considered as potential confounders. We included sex, age, education level, smoking, alcohol intake, physical activity, sedentary time, BMI, waist circumference and visceral fat area as potential confounders. We estimated a multivariable logistic regression model between the potential confounders and NAFLD in order to assess individual propensity scores for the presence of NAFLD. The propensity score is a balancing score [34]: by conditioning on the propensity score, the distribution of observed confounders should be similar between subjects with and without NAFLD. Achieved balance of the confounders between the risk factor groups (NAFLD vs. non NAFLD) by the propensity score approach can be demonstrated [34].

We considered the following covariates as *metabolic risk factors* for each outcome, including conceivable intermediates that might have been influenced/modified by the presence of NAFLD: diabetes status (3 categories: no diabetes, IFG, diabetes), hypertension, high LDL cholesterol, low HDL cholesterol, triglycerides, hsCRP and insulin. The continuous variables included as potential confounders and metabolic risk factors were categorized with cut-offs or corresponding to quartiles.

We employed logistic regression to examine the associations between NAFLD and the two dichotomous outcomes (CACS and carotid plaques, >0 vs. 0) and a generalized linear model to examine the association between NAFLD and IMT (log-transformed outcome data). Firstly, NAFLD was included as the only covariate (Model 1). Confounding was thereafter controlled for by including the propensity score as a covariate (Model 2). We checked for the model relationship between the propensity score and the outcome (linear relationship with log-odds for CACS>0 and plaque>0, respectively; linear relationship with ln[IMT]). For comparison, we also controlled for confounding by a conventional approach by including the potential confounders as covariates (rather than the propensity score as a covariate). Finally, we added the other metabolic risk factors to the set of covariates (Model 3). Inclusion of intermediates may yield over-adjustment; therefore, it is of interest to report estimates provided by Models 2 and 3, respectively. We repeated the analyses based on Models 1 and 2 for subgroups according to each factor among 7 metabolic risk factors. We also analyzed subgroups according to a summary measure: the number of metabolic risk factors present (0 to 7, presence of diabetes *or* IFG was counted as 1). Heterogeneity of association estimates across subgroups was examined by including in Model 2 the subgroup variable and an interaction term between this variable and NAFLD; a significant interaction term can be interpreted as significant heterogeneity of association across the subgroups. Statistical analyses were performed using SPSS for Windows, version 23.

## Results

NAFLD was more common in men than in women (15.4% and 5.8%, respectively). Subjects with NAFLD were more obese and had more of obesity-related metabolic aberrations than subjects without NAFLD, including impaired glucose homeostasis, hypertension, high triglyceride levels, low HDL and high CRP (Table 1). In the propensity score model with potential confounders, BMI and visceral adipose tissue were significantly associated with NAFLD (S2 Table). Apart from sex and body fat variables, there was no major influence by the other potential confounders in the propensity score model (i.e. age, education, smoking, alcohol, physical activity and sedentary time). The estimated propensity scores for NAFLD, conditional on the potential confounders, turned out as an appropriate variable for confounding adjustment. By conditioning on the propensity score, we achieved good balance of each confounder between subjects with and without NAFLD (S2 Table).

**Table 1:** Characteristics of overall study population and comparison between subjects with and without non-alcoholic fatty liver disease (NAFLD).

	Overall (n = 1015)	No NAFLD (n = 909)	NAFLD (n = 106)	p value ^a^
Female [n (%)]	520 (51.2)	490 (53.9)	30 (28.3)	<0.0001
Age (years)	57.6 (4.4)	57.5 (4.4)	58.3 (4.5)	0.082
Secondary school or above [n (%)]	822 (81.5)	744 (82.4)	78 (74.3)	0.043
*Lifestyle*				
Current smoker [n (%)]	141 (13.9)	127 (14.0)	14 (13.2)	0.82
Alcohol use (g/day)	6.8 (6.6)	6.7 (6.3)	7.9 (8.3)	0.87
Physical activity time (%)^b^	5.2 (2.6)	5.3 (2.6)	4.6 (2.3)	0.049
Sedentary time (%)	69.2 (7.6)	69.0 (7.6)	70.8 (7.0)	0.084
*Body fat*				
Obesity [n (%)]	226 (22.3)	171 (18.8)	55 (51.9)	<0.0001
BMI (kg/m^2^)	27.3 (4.5)	26.9 (4.2)	31.4 (4.8)	<0.0001
Waist circumference (cm)	95.3 (12.8)	93.8 (12)	108.4 (12)	<0.0001
SAT (cm^2^)	269.5 (113.3)	264.3 (111.2)	313.8 (122)	<0.0001
VAT (cm^2^)	165.8 (87.3)	154 (79)	267 (89.9)	<0.0001
Liver attenuation (HU)	53.3 (10.3)	56 (6.3)	30.2 (9.2)	
*Blood pressure*				
Hypertension [n (%)]	313 (30.8)	255 (28.1)	58 (54.7)	<0.0001
Systolic blood pressure (mmHg)	124.4 (16.9)	123 (15.9)	136.9 (19.5)	<0.0001
Diastolic blood pressure (mmHg)	74.3 (9.2)	73.6 (8.9)	80.8 (9.3)	<0.0001
*Glucose regulation*				
Diabetes [n (%)]	73 (7.3)	43 (4.8)	30 (28.3)	<0.0001
Impaired fasting glucose [n (%)]	145 (14.5)	114 (12.7)	31 (29.2)	<0.0001
Glucose (mmol/L)	5.9 (1.3)	5.7 (1.1)	7.0 (2.2)	<0.0001
Hemoglobin A1c (mmol/mol)	36.4 (7.3)	35.7 (6.1)	42.1 (12.6)	<0.0001
Insulin (mU/L)	8.8 (7.9)	7.8 (6.1)	17.3 (14.1)	<0.0001
*Lipids*				
Total cholesterol (mmol/L)	5.8 (1.0)	5.8 (1.0)	5.7 (1.2)	0.41
LDL cholesterol (mmol/L)	3.8 (0.9)	3.8 (0.9)	3.8 (1.1)	0.48
HDL cholesterol (mmol/L)	1.7 (0.5)	1.7 (0.5)	1.4 (0.4)	<0.0001
Triglycerides (mmol/L)	1.3 (0.8)	1.3 (0.7)	2.0 (1.2)	<0.0001
*Inflammation*				
hsCRP (mg/L)	2.3 (3.6)	2.2 (3.7)	3.1 (2.6)	<0.0001

Continuous variables are given as mean (SD) unless otherwise indicated.

^a^ Differences in characteristics between subjects with and without NAFLD were determined using chi-square test for dichotomous variables and Mann-Whitney U test for continuous variables.

^b^ Measured using accelerometer as percent of valid wear time spent in moderate to vigorous physical activity (MVPA).

Abbreviations: NAFLD, non-alcoholic fatty liver disease; BMI, body mass index; SAT, subcutaneous adipose tissue; VAT, visceral adipose tissue; HU, Hounsfield Units; LDL, low density lipoprotein; HDL, high density lipoprotein; hsCRP, high sensitivity C-reactive protein.

NAFLD was significantly associated with CACS after confounder adjustment using propensity score in Model 2 (OR 2.11, 95% CI 1.32–3.39) (Table 2). The association between NAFLD and CACS remained significant after additional adjustment for metabolic risk factors (OR 1.77, 95% CI 1.07–2.94 [Model 3]). In contrast, there were no significant associations between NAFLD and the two measures of carotid atherosclerosis, i.e. carotid plaques (Model 2: OR 1.21, 95% CI 0.77–1.90) and IMT (Model 2: ratio between the means for the NAFLD and no NAFLD groups 1.04, 95% CI 0.99–1.08) (Table 2). The conventional approach to controlling for confounding yielded a marginally higher OR estimate between NAFLD and CACS (2.22, 95% CI 1.36–3.64), as well as between NAFLD and carotid plaques (1.25, 95% CI 0.79–1.97), and a similar IMT mean ratio (1.04, 95% CI 1.00–1.08), as compared with the estimates by Model 2 presented above.

**Table 2:** Associations between NAFLD and outcome variables, with account for propensity score (adjustment for confounders) and metabolic risk factors.

Outcome	NAFLD (n = 106)	No NAFLD (n = 909)	Association^a^ Model 1^b^	Association^a^ Model 2^c^	Association^a^ Model 3^d^
CACS>0 [% (n/N)]	69 (73/106)	38 (344/909)	3.63 (2.36–5.60)	**2.11 (1.32–3.39)**	**1.77 (1.07–2.94)**
Plaques>0 [% (n/N)]	60 (64/106)	55 (498/909)	1.26 (0.83–1.90)	1.21 (0.77–1.90)	1.20 (0.74–1.95)
IMT [mean ±SD]	0.83 ±0.21^e^	0.76 ±0.14^e^	1.07 (1.03–1.12)	1.04 (0.99–1.08)	1.02 (0.98–1.07)

^a^ Odds ratio (95% confidence interval) for the dichtomized outcomes (CACS and carotid plaques). For the continuous IMT outcome, the ratio between the means for the NAFLD and no NAFLD groups (95% confidence interval).

^b^ Model 1: without adjustment.

^c^ Model 2: with account for propensity score, i.e. adjustment for the potential confounders (listed in Fig 2).

^d^ Model 3: with additional adjustment for metabolic risk factors including diabetes status (no diabetes, IFG, diabetes), hypertension (no, yes), LDL cholesterol (<4 vs. ≥4 mmol/L), HDL cholesterol (≥1.3 vs. <1.3 mmol/L for women; ≥1.0 vs. <1.0 for men), triglycerides (<1.7 vs. ≥1.7 mmol/L), hsCRP (<5 vs. ≥5 mg/L) and insulin (<20 vs. ≥20 mU/L).

^e^ No. of observations (due to misssing data for IMT): n = 102 among subjects with NAFLD and n = 896 among subjects without NAFLD.

**Fig 3:**
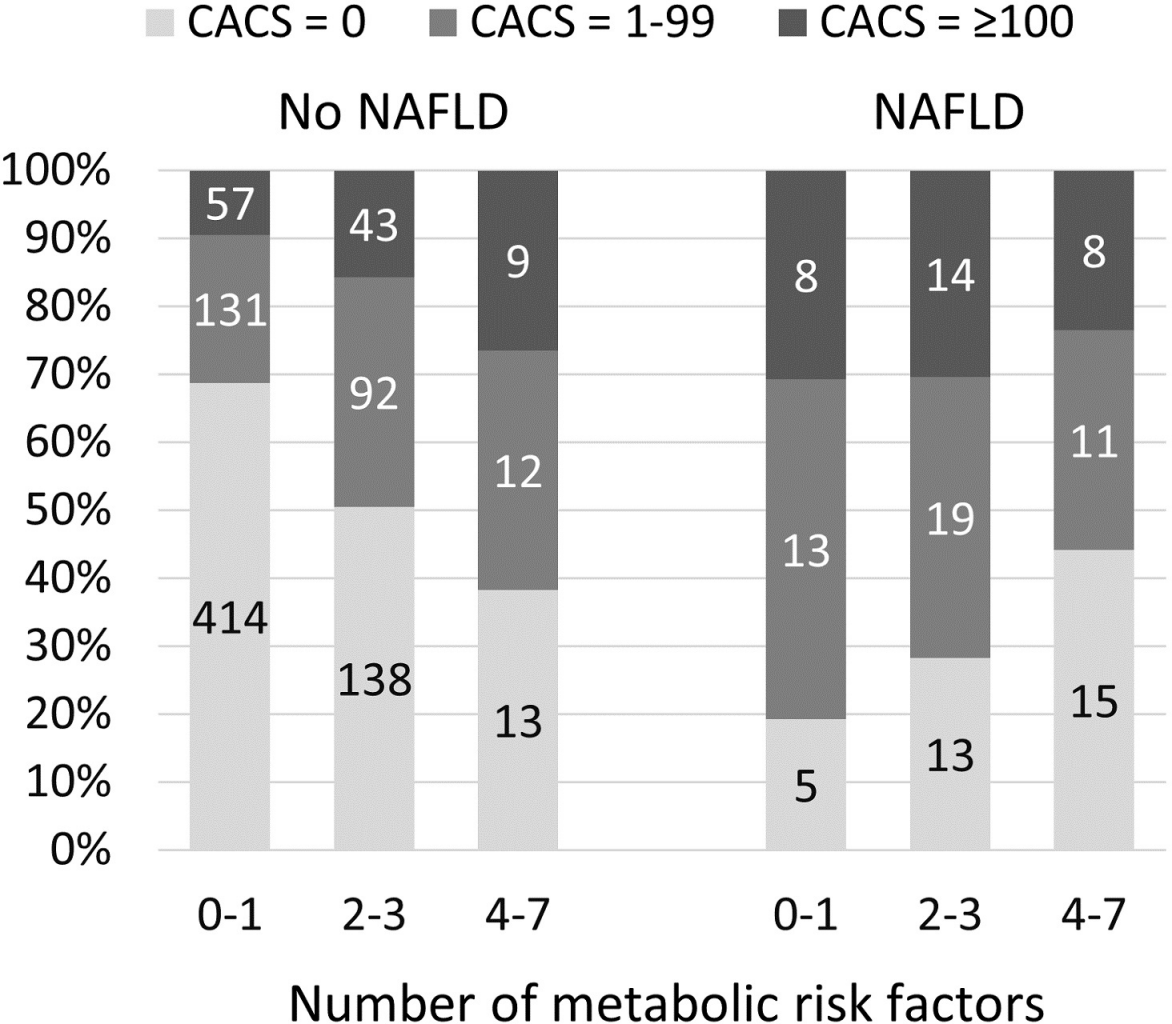
Proportions (in percent) of subjects with CACS = 0, 1–99, and ≥100 according to NAFLD and metabolic status. Numbers within bars depict the number of subjects within each category.

Since there was no association between NAFLD and measurements of carotid atherosclerosis, we did not proceed with subgroup analyses for these outcomes. Subgroup analyses regarding CACS indicated that the association between NAFLD and CACS was modified by the metabolic risk factors (Table 3). When the population was grouped based on the presence/absence of each metabolic risk factor, associations between NAFLD and CACS were consistently more marked when the risk factor was absent. The estimates (with adjustments for confounders) differed significantly across the subgroups according to hypertension, HDL cholesterol and triglycerides but not significantly across the subgroups not according to diabetes status, LDL cholesterol, hsCRP and insulin. Overall, the strongest association between NAFLD and CACS was observed in subjects with few other metabolic risk factors (612 subjects [60% of the total cohort] with 0 or 1 of the 7 predefined risk factors; OR 5.94, 95% CI 2.13–16.6), suggesting that the association between NAFLD and coronary artery calcification is most marked in subjects with few metabolic risk factors. The proportions of subjects with positive CACS according to NAFLD and metabolic status are illustrated in Fig 3. In this figure the data was further divided into subjects with CACS 1–99 and ≥100, illustrating how odds ratios are driven by both categories.

**Table 3:** Associations between NAFLD and positive coronary artery calcification score (CACS) in subgroups of subjects according to their metabolic risk factors.

*Subgroup*	CACS>0 among NAFLD, % (n)	CACS>0 among No NAFLD, % (n)	Model 1^b^ OR (95% CI)^a^	Model 2^c^ OR (95% CI)^a^
By diabetes status, *p* = 0.24^d^				
No diabetes or IFG	67 (30/45)	34 (259/752)	3.81 (2.01–7.20)	**2.72 (1.40–5.29)**
IFG	71 (22/31)	52 (59/114)	2.28 (0.97–5.37)	1.28 (0.49–3.36)
Diabetes	70 (21/30)	60 (26/43)	1.53 (0.57–4.11)	0.90 (0.29–2.85)
By hypertension, *p* = 0.04^d^				
No	71 (34/48)	31 (206/654)	5.28 (2.77–10.1)	**3.53 (1.76–7.09)**
Yes	67 (39/58)	54 (138/255)	1.74 (0.95–3.18)	1.09 (0.56–2.10)
By LDL cholesterol (mmol/L)^e^, *p* = 0.24^d^				
<4	70 (38/54)	35 (185/522)	4.33 (2.35–7.97)	**2.37 (1.22–4.58)**
≥4	67 (35/52)	41 (159/385)	2.93 (1.58–5.41)	2.00 (1.00–3.99)
By HDL cholesterol (mmol/L)^e^, *p* = 0.02^d^				
≥1.3 women, ≥1.0 men	74 (61/81)	38 (316/840)	4.74 (2.83–7.94)	**2.79 (1.61–4.85)**
<1.3 women, <1.0 men	52 (13/25)	42 (28/67)	1.51 (0.60–3.80)	0.96 (0.31–2.98)
By triglycerides (mmol/L)^f^, *p* = 0.002^d^				
<1.7	78 (40/51)	35 (254/729)	6.80 (3.43–13.5)	**4.46 (2.17–9.16)**
≥1.7	60 (33/55)	51 (90/177)	1.45 (0.78–2.68)	0.89 (0.45–1.76)
By hsCRP (mg/L)^e^, *p* = 0.08^d^				
<5	72 (63/88)	37 (307/825)	4.25 (2.62–6.90)	**2.53 (1.50–4.28)**
≥5	56 (10/18)	45 (37/82)	1.52 (0.55–4.24)	0.80 (0.25–2.58)
By insulin (mU/L)^e^, *p* = 0.49^d^				
<20	68 (50/74)	38 (331/882)	3.47 (2.09–5.75)	**2.27 (1.33–3.88)**
≥20	72 (23/32)	52 (13/25)	2.36 (0.78–7.08)	1.15 (0.31–4.22)
By no. of metabolic risk factors present^e^^,^^g^, *p* = 0.002^d^				
0–1	78 (18/23)	32 (187/589)	7.74 (2.83–21.1)	**5.94 (2.13–16.6)**
2–3	74 (35/47)	48 (133/279)	3.20 (1.60–6.42)	**2.33 (1.12–4.87)**
4–7	57 (20/36)	63 (24/38)	0.73 (0.29–1.85)	0.31 (0.10–1.01)

^a^ Odds ratio (95% confidence interval).

^b^ Model 1: without adjustment.

^c^ Model 2: with account for propensity score, i.e. adjustment for confounders.

^d^ Test of homogeneity of OR across the the subgroups based on Model 2 (i.e. with adjustment for confounders).

^e^ Missing data for 2 persons.

^f^ Missing data for 3 persons.

^g^ Presence of diabetes *or* IFG was counted as 1.

## Discussion

The aim of this study was to estimate the relationship between NAFLD and measures of atherosclerotic cardiovascular disease (ASCVD) and to determine to what extent such relationships are modified by metabolic risk factors. Importantly, we wanted to address these questions without the influence of age, gender, obesity and other potential confounders. By using the propensity score model we balanced the NAFLD and non NAFLD groups with regards to adiposity and potential confounders, and subsequently investigated the association between NAFLD and ASCVD in relation to the overall burden of metabolic risk factors. For classification according to metabolic status we used variables that are known to be associated with obesity and/or ASCVD, including diabetes/IFG, hypertension, hyperinsulinemia, dyslipidemia (low HDL, high LDL or high triglycerides) and elevated hsCRP. We found that the presence of NAFLD is associated with a two-fold increased risk of coronary artery calcification in the overall study population after confounder adjustment. Furthermore, the association between NAFLD and CACS remained significant after adjustment for all of the metabolic risk factors, and subgroup analyses showed that the effect estimate was primarily driven by the metabolically healthy part of the population. The strong association between NAFLD and CACS was contrasted by a lack of association between NAFLD and measures of carotid atherosclerosis (i.e. carotid plaques and IMT).

Our finding that NAFLD is associated with coronary artery disease (CAD) is consistent with several previous reports, including population-based cohorts such as the Jackson Heart study [35], the Framingham Heart study [11], the Multi-Ethnic Study of Atherosclerosis (MESA) [4], and the Coronary Artery Risk Development in Young Adults (CARDIA) study [17]. Similar to our study, they used CT to assess liver fat and their classification of CAD was based on CT-assessed coronary calcium scores. CACS scanning is a well-established method for identifying subclinical coronary atherosclerosis that has been extensively validated with regards to other measures of CAD [36]. Also, CACS has been shown to be a strong predictor of ASCVD events in prospective cohort data: in the MESA cohort, a CACS of 1–100 conferred a 4-fold higher risk for events, and a CACS of >100 conferred a 7-fold higher risk for events, compared with a CACS of 0 [37]. Thus, a two-fold increased risk of coronary artery calcification has considerable implications for ASCVD risk prediction.

Several previous studies reported NAFLD to be an independent predictor of CACS [4, 6–16], whereas in the CARDIA study the association between NAFLD and CACS was attenuated after adjustment for established cardiovascular risk factors, including obesity, dyslipidemia, hypertension, diabetes and smoking status [17]. The CARDIA study is quite similar to our study, both being population-based studies that use CT for the assessment of liver and visceral fat and CACS as outcome measure. However, CARDIA is a USA-based cohort consisting of 50% black subjects and it is possible that differences in ethnicity between cohorts may have played a role. Our multivariable models included established cardiovascular risk factors as well as additional variables that that are less commonly represented in previous reports, such as insulin and CRP. Even with these extensive adjustments, the association persisted for NAFLD and CACS. This observation, together with the higher relative risk seen in the metabolically healthy subpopulation, adds to the already existing evidence that the link between NAFLD and CAD is not solely mediated by the classical metabolic risk factors that are commonly associated with obesity and/or ASCVD.

The strongest association between NAFLD and CACS was observed in subjects with few metabolic risk factors according to diabetes status, hypertension, LDL, HDL, triglycerides, hsCRP and insulin. In this subgroup we observed that subjects with NAFLD had as much as a 6-fold increased risk of coronary artery calcification as compared to those not having NAFLD. Previous studies in metabolically healthy subpopulations are scarce and therefore our findings should be confirmed in other cohorts before we can firmly conclude that the relative risk is of this magnitude. The present findings in the SCAPIS pilot cohort may be tested in the larger SCAPIS cohort (n = 30,000) within a few years from now [20].

In contrast to the strong association between NAFLD and CACS, we did not observe any convincing association with measures of carotid atherosclerosis in this population. The evidence of a positive correlation between NAFLD and carotid atherosclerosis in the literature mainly comes from hospital-based case-control studies and from Asian community-based studies using ultrasonography to assess liver steatosis [3, 38]. Fewer studies have compared CT-assessed liver fat with measures of carotid atherosclerosis. The Multicultural Community Health Assessment trial reported that associations between CT-assessed liver fat and measures of subclinical carotid atherosclerosis were attenuated after adjustment for anthropometric measures [39]. Two studies addressing this question using ^1^H-MRS to measure liver fat, both of them conducted in T2D patients, did not confirm a positive correlation: Petit *et al* reported that NAFLD was not associated with carotid IMT [40] and Loffroy *et al* found that liver fat was content was negatively associated with carotid plaque burden [41]. Taken together, data on this matter has not been entirely consistent, possibly due to differences between studies with regards to imaging modality, ethnicity and/or metabolic burden. Furthermore, the underlying cause of hepatic fat accumulation may be an important determinant of the relationship between NAFLD and atherosclerosis. It is now recognized that some genetic forms of NAFLD associate with a relative protection against metabolic dysregulation and atherosclerosis due to single nucleotide polymorphisms in the genes patatin-like phospholipase domain-containing protein-3 (PNPLA3) and transmembrane 6 superfamily member 2 protein (TM6SF2) [42, 43]. Since genotypes are unknown in this cohort, we cannot exclude the possibility that associations between NAFLD and carotid outcome variables have been weakened by the presence of such genetic variants, although unlikely considering the strong correlation between NAFLD and CACS.

Several mechanisms through which NAFLD promotes ASCVD have been proposed. There is now convincing evidence that NAFLD is tightly linked to the cardiometabolic risk factors studied here, i.e. glucose dysregulation, dyslipidemia, hypertension and elevated CRP [4, 44]. However, for the group of patients who do not display these classical risk factors, alternative explanations for the strong association between NAFLD and coronary artery calcification need to be sought. A growing body of data shows that NAFLD promote dysfunctional secretion of a wide range of fatty acids, lipoproteins, proinflammatory cytokines, enzymes and microRNAs with pro-atherogenic properties [3] [45]. Thus, clinical studies that include an even more detailed and also a broader range of potential modifiers and mediators are warranted to evaluate the underlying mechanism. As an example, the dyslipidemia in NAFLD has a complexity that is not fully captured by the standard lipid panel such as altered levels of pro- and anti-atherogenic subpopulations of LDL and HDL cholesterol [46]. It is also important to note that coronary and carotid atherosclerosis have been measured with different modalities representing different aspects of ASCVD. Given the significant correlation with coronary artery calcification, but not with carotid IMT or plaques, it might be of relevance to study if relationships are modified by factors involved in ectopic calcification. Fetuin-A and osteopontin are two examples of proteins involved in coronary artery calcification that are altered by NAFLD and thus interesting to study further in this context [47–49].

An important strength of the present study is the well-characterized population-based cohort which made it possible to take into account a wide range of covariates in the analyses. Unlike most previous studies we have characterized the relationship between NAFLD and subclinical atherosclerosis in both coronary and carotid arteries. By studying both vascular regions in the same population using the same statistical approach it becomes apparent that NAFLD is more strongly linked to coronary than to carotid atherosclerosis. Our statistical approach using propensity score was primarily chosen to allow for separate handling of confounders and metabolic risk factors including conceivable intermediates that might have been influenced and/or modified by the presence of NAFLD. The propensity score method provides transparency of data analysis, since researchers can thereby assess whether observed confounding has been adequately balanced between the primary risk factor (in this case NAFLD). By comparison with a conventional approach to controlling for confounding (including the potential confounders as covariates), we demonstrated somewhat more pronounced downward adjustments of the effect estimates by the propensity score method.

Some limitations warrant mention. The study is observational and cross-sectional, which limits the ability to infer temporal or causal relationships. This is a population-based cohort and therefore the number of subjects with pronounced ASCVD is relatively low. The data on history of liver disease from other causes were obtained from medical records and not an extensive laboratory screening, and we may therefore have included some subjects with secondary steatosis. The NAFLD diagnosis was defined based only on CT examinations and was not biopsy verified since such invasive procedures would not be feasible in a large population-based cohort. Thus, we were not able to distinguish simple steatosis from NASH. CT is a well-established method to noninvasively quantify liver fat and the method has been used in several prior epidemiologic cohorts to assess NAFLD status [4, 7, 11–13, 16, 17, 35]. We applied the commonly used liver attenuation cut-off of 40 HU to define NAFLD, which corresponds to approximately 30% steatosis at histological analysis [23] and approximately 13–15% liver fat content as measured by MRS/MRI proton-density fat fraction (PDFF) methods [50]. This cut-off provides a high specificity for moderate to severe NAFLD but is relatively insensitive to detect mild steatosis [51]. Thus the results are primarily relevant for moderate to severe steatosis. Obesity has a broad impact on human metabolism and thus there are probably additional variables that may be relevant in the assessment of metabolic status. However, we believe that our spectrum of variables covers the main risk factors and features of the metabolic syndrome that are commonly obtained in the clinical setting.

In conclusion, our main findings were that NAFLD was significantly associated with our measure of subclinical CAD (i.e. CACS) and that the risk increase was most prominent in subjects with few metabolic risk factors. The results suggest that screening for subclinical CAD may be relevant for all NAFLD patients regardless of their metabolic status. Key questions for future research are to elucidate the mechanisms that link NAFLD to CAD, and to test if NAFLD-treatments can reduce CAD risk.

## Supporting information

S1 Table Potential confounders in relation to NAFLD, presented as relative frequencies (%) and odds ratios (OR) with 95% confidence intervals (CI). (DOCX)

S2 Table Achieved confounder balance between the groups with (+) and without (-) NAFLD by propensity score (PS) stratification, presented as relative frequencies (%) for each confounder conditional on NAFLD group. (DOCX)
