# Supplementary material for: Non-alcoholic fatty liver disease is a strong predictor of coronary artery calcification in metabolically healthy subjects: A cross-sectional, population-based study in middle-aged subjects
Source: PLoS One. 2018 Aug 22;13(8):e0202666. doi: 10.1371/journal.pone.0202666 (PMC6105021; doi:10.1371/journal.pone.0202666)
Supplement: S1 Table — (DOCX) [file pone.0202666.s001.docx]

**S1 Table.** Potential confounders in relation to NAFLD, presented as relative frequencies (%) and odds ratios (OR) with 95% confidence intervals (CI).

| **Potential confounder** | **NAFLD+ (n=106)** | **NAFLD- (n=909)** | **Univariable model OR (95% CI)** | **Multivariable model^a^ OR (95% CI)** |  |
| --- | --- | --- | --- | --- | --- |
| *Sex* | | | | |  |
| Women | 28 | 54 | 1.0 (Reference) | 1.0 (Reference) |  |
| Men | 72 | 46 | 2.96 (1.90 – 4.61) | 3.16 (1.93 – 5.18) |  |
| *Age (years)* | | | | | |
| 50 – 54 | 28 | 33 | 1.0 (Reference) | 1.0 (Reference) |  |
| 55 – 59 | 29 | 35 | 0.97 (0.57 – 1.64) | 0.94 (0.52 – 1.68) |  |
| 60 – 65 | 43 | 33 | 1.50 (0.92 – 2.44) | 1.31 (0.76 – 2.28) |  |
| *Education* | | | | | |
| University | 33 | 37 | 1.0 (Reference) | 1.0 (Reference) |  |
| Secondary school | 41 | 44 | 1.03 (0.65 – 1.65) | 0.81 (0.48 – 1.37) |  |
| Primary school or  missing (n=7) | 26 | 18 | 1.64 (0.97 – 1.64) | 1.08 (0.59 – 1.98) |  |
| *Smoking* | | | | |  |
| Non smoker |  |  | 1.0 (Reference) | 1.0 (Reference) |  |
| Current smoker or missing (n=4) | 13 | 14 | 0.90 (0.50 – 1.63) | 0.87 (0.45 – 1.68) |  |
| *Alcohol intake (g/day)* | | | | | |
| <10 women, <20 men | 76 | 78 | 1.0 (Reference) | 1.0 (Reference) |  |
| ≥10 women, ≥20 men | 11 | 10 | 1.10 (0.58 – 2.10) | 0.92 (0.45 – 1.89) |  |
| missing (n=119) | 12 | 12 | 1.07 (0.60 – 1.99) | 0.79 (0.39 – 1.59) |  |
| *Physical activity (% time in MVPA)* | | | | | |
| ≥5 | 31 | 44 | 1.0 (Reference) | -^b^ |  |
| <5 | 45 | 45 | 1.42 (0.89 – 2.26) | - |  |
| missing (n=133) | 24 | 12 | 2.29 (1.40 – 3.74) | 1.71 (0.96 – 3.06) |  |
| *Sedentary time (%)* | | | | | |
| ≤75 | 58 | 69 | 1.0 (Reference) | -^b^ |  |
| >75 | 18 | 19 | 1.09 (0.63 – 1.87) | - |  |
| missing (n=133) | 24 | 12 | 2.29 (1.40 – 3.74) | 1.71 (0.96 – 3.06) |  |
| *BMI* | | | | | |
| <25 | 2 | 35 | 1.0 (Reference) | 1.0 (Reference) |  |
| 25 – <30 | 46 | 46 | 18.2 (4.40 – 75.6) | 6.15 (1.38 – 27.3) |  |
| 30+ | 52 | 19 | 50.7 (12.2 – 210) | 10.3 (2.13 – 50.2) |  |
| *Waist circumference (cm)* | | | | |  |
| ≤88 women, ≤102 men | 25 | 59 | 1.0 (Reference) | 1.0 (Reference) |  |
| >88 women, >102 men | 75 | 41 | 4.46 (2.81 – 7.08) | 0.95 (0.49 – 1.83) |  |
| *Visceral fat area (cm^2^)* | | | | | |
| 1^st^ and 2^nd^ quartile: 0-116  women, 0-195 men | 8 | 55 | 1.0 (Reference) | 1.0 (Reference) |  |
| 3^rd^ quartile: >116-165  women, >195 men | 27 | 25 | 7.09 (3.30 – 15.2) | 4.79 (2.09 – 10.8) |  |
| 4^th^ quartile: >165 women,  >256 men | 64 | 20 | 20.2 (9.87 – 41.3) | 9.86 (4.24 – 22.9) |  |

^a^ The multivariable model yielded the propensity scores (i.e., the predicted probabilities based on the multivariable logistic regression model; baseline odds = 0.0077)

^b^ Absence of data on physical activity and sedentary time (both variables missing for 133 persons) was significantly associated with the presence of NAFLD, whereas level of physical activity and level of sedentary time were not notably associated with NAFLD in multivariable models. Only the indicator physical activity/sedentary time missing were therefore included in the multivariable model.
