# Supplementary material for: Non-alcoholic fatty liver disease is a strong predictor of coronary artery calcification in metabolically healthy subjects: A cross-sectional, population-based study in middle-aged subjects
Source: PLoS One. 2018 Aug 22;13(8):e0202666. doi: 10.1371/journal.pone.0202666 (PMC6105021; doi:10.1371/journal.pone.0202666)
Supplement: S2 Table — (DOCX) [file pone.0202666.s002.docx]

**S2 Table.** Achieved confounder balance between the groups with (+) and without (-) NAFLD by propensity score (PS) stratification, presented as relative frequencies (%) for each confounder conditional on NAFLD group.^a^

|  | Total | | PS 1 | | PS 2 | | PS 3 | | PS 4 | |
| --- | --- | --- | --- | --- | --- | --- | --- | --- | --- | --- |
| NAFLD | + | - | + | - | + | - | + | - | + | - |
| *Confounder* | | | | | | | | | | |
| Sex (men) | 72 | 46 | 46 | 39 | 56 | 59 | 85 | 80 | 100 | 100 |
| Age (years) | | | | | | | | | | |
| 50 – 54 | 28 | 33 | 38 | 34 | 15 | 33 | 26 | 24 | 35 | 24 |
| 55 – 59 | 29 | 35 | 35 | 36 | 37 | 30 | 19 | 28 | 27 | 21 |
| 60 – 65 | 43 | 33 | 27 | 30 | 48 | 38 | 56 | 48 | 38 | 55 |
| Education | | | | | | | | | | |
| University | 33 | 37 | 31 | 38 | 41 | 44 | 26 | 29 | 35 | 38 |
| Secondary school | 41 | 44 | 50 | 46 | 48 | 38 | 41 | 42 | 23 | 31 |
| Primary school or  missing (n=7) | 26 | 18 | 19 | 16 | 11 | 19 | 33 | 29 | 42 | 31 |
| Current smoker | 13 | 14 | 15 | 15 | 15 | 14 | 7 | 11 | 15 | 14 |
| Alcohol intake (g/day) | | | | | | | | | | |
| <10 women, <20 men | 76 | 78 | 81 | 78 | 74 | 80 | 78 | 77 | 73 | 83 |
| ≥10 women, ≥20 men | 11 | 10 | 4 | 11 | 11 | 8 | 15 | 11 | 15 | 7 |
| missing | 12 | 12 | 15 | 12 | 15 | 12 | 7 | 11 | 11 | 10 |
| Physical activity (% time in MVPA) | | | | | | | | | | |
| ≥5 | 31 | 44 | 54 | 46 | 41 | 39 | 15 | 37 | 15 | 17 |
| <5 | 45 | 45 | 46 | 44 | 37 | 55 | 70 | 46 | 27 | 34 |
| missing | 24 | 12 | 0 | 10 | 22 | 6 | 15 | 18 | 58 | 48 |
| Sedentary time (%) | | | | | | | | | | |
| ≤75 | 58 | 69 | 85 | 71 | 67 | 78 | 56 | 52 | 27 | 31 |
| >75 | 18 | 19 | 15 | 18 | 11 | 16 | 30 | 30 | 15 | 21 |
| missing | 24 | 12 | 0 | 10 | 22 | 6 | 15 | 18 | 58 | 48 |
| BMI | | | | | | | | | | |
| <25 | 2 | 35 | 8 | 43 | 0 | 0 | 0 | 0 | 0 | 0 |
| 25 – <30 | 46 | 46 | 61 | 48 | 56 | 48 | 56 | 48 | 12 | 14 |
| 30+ | 52 | 19 | 31 | 10 | 44 | 52 | 44 | 52 | 88 | 86 |
| Large waist circumference  (>88 cm women, >102 cm men) | 75 | 41 | 54 | 32 | 70 | 72 | 82 | 79 | 96 | 97 |
| Visceral fat area (cm^2^) | | | | | | | | | | |
| 1^st^ and 2^nd^ quartile: 0-116  women, 0-195 men | 8 | 55 | 35 | 67 | 0 | 0 | 0 | 0 | 0 | 0 |
| 3^rd^ quartile: >116-165  women, >195 men | 27 | 25 | 38 | 24 | 59 | 55 | 11 | 21 | 0 | 0 |
| 4^th^ quartile: >165 women,  >256 men | 64 | 20 | 27 | 9 | 41 | 45 | 89 | 79 | 100 | 100 |

^a^ The PS strata correspond to the PS quartiles for the 106 individuals with NAFLD (+), including the following numbers of persons: 26 (with NAFLD) + 737 (without NAFLD) in stratum 1 (PS ≤ 0.156); 27 + 64 in stratum 2; (0.156 < PS ≤ 0.223) 27 + 79 in stratum 3 (0.223 < PS ≤ 0.383); and 26 + 29 in stratum 4 (PS > 0.383). The propensity scores were obtained from t
